# Association of microRNAs and pathologic response to preoperative chemotherapy in triple negative breast cancer: preliminary report

**DOI:** 10.1007/s11033-014-3140-7

**Published:** 2014-01-29

**Authors:** Agnieszka Kolacinska, Jan Morawiec, Wojciech Fendler, Beata Malachowska, Zbigniew Morawiec, Janusz Szemraj, Zofia Pawlowska, Dipanjan Chowdhury, Young Eun Choi, Robert Kubiak, Lukasz Pakula, Izabela Zawlik

**Affiliations:** 1 Department of Surgical Oncology, Copernicus Memorial Hospital, Cancer Center, Paderewskiego 4, 93-509 Lodz, Poland; 2 Department of General and Colorectal Surgery, Medical University of Lodz, Hallera Sq. 1, 90-647 Lodz, Poland; 3 Department of Pediatrics, Oncology, Hematology and Diabetology, Medical University of Lodz, Sporna 36/50, 91-738 Lodz, Poland; 4 Department of Medical Biochemistry, Medical University of Lodz, Mazowiecka 6/8, 92-215 Lodz, Poland; 5 Central Laboratory Corelab, Medical University of Lodz, Mazowiecka 6/8, 92-215 Lodz, Poland; 6 Department of Radiation Oncology, Dana-Farber Cancer Institute, Harvard Medical School, 450 Brookline Av, Boston, MA 02215 USA; 7 Department of Pathology, Medical University of Lodz, Pabianicka 62, 93-513 Lodz, Poland; 8 Department of Anesthesiology, Copernicus Memorial Hospital, Pabianicka 62, 93-513 Lodz, Poland; 9 Department of Medical Genetics, Institute of Nursing and Health Sciences, Medical Department, University of Rzeszow, Rejtana 16c, 35-959 Rzeszow, Poland

**Keywords:** Breast cancer, microRNA, Pathologic response

## Abstract

Triple negative breast cancer (TNBC) has caught the attention of oncologists worldwide because of poor prognosis and paucity of targeted therapies. Gene pathways have been widely studied, but less is known about epigenetic factors such as microRNAs (miRNAs) and their role in tailoring an individual systemic and surgical approach for breast cancer patients. The aim of the study was to examine selected miRNAs in TNBC core biopsies sampled before preoperative chemotherapy and the subsequent pathologic response in mastectomy or breast conservation specimens. Prior to treatment, core needle biopsies were collected from 11 female patients with inoperable locally advanced TNBC or large resectable tumors suitable for down-staging. In all 11 TNBC core biopsies we analyzed 19 miRNAs per sample: 512, 190, 200, 346, 148, 449, 203, 577, 93, 126, 423, 129, 193, 182, 136, 135, 191, 122 and 222 (miRCURY LNA™ Universal RT microRNA polymerase chain reaction Custom Pick & Mixpanels). The Wilcoxon signed-rank test was used to compare related samples. Ingenuity pathway analysis was used to evaluate potential functional significance of differentially expressed miRNAs. Statistical analysis showed that 3 of 19 miRNAs differed in relation to pathologic response i.e. good versus poor. These differences failed to reach statistical significance, although a trend was observed (*p* = 0.06). Among these miRNAs, we identified—miR-200b-3p, miR-190a and miR-512-5p. In summary, our results indicate that higher miR-200b-3p, higher miR-190a and lower miR-512-5p expression levels in core biopsies sampled from TNBC patients may be associated with better pathologic response to chemotherapy and the increased feasibility of breast conserving surgery in these patients. Although these results were from a small cohort, they provide an important basis for larger, prospective, multicenter studies to investigate the potential role of miRNAs in neoadjuvant setting.

## Introduction

Triple negative breast cancer (TNBC) has caught the attention of oncologists worldwide because of poor prognosis and paucity of targeted therapies [1]. The preoperative setting is an optimal model for in-depth research on TNBC [2, 3]. Firstly there is the opportunity to fast-track testing of novel biomarkers and therapeutic agents, and secondly pathologic complete response (pCR) to neoadjuvant systemic therapy is a valid surrogate for better outcome in TNBC [4]. Multiple studies have demonstrated marked sensitivity of TNBC to chemotherapy compared with luminal A breast cancer [1–4]. Nevertheless 66–72 % of TNBC patients fail to achieve pCR after treatment with preoperative anthracycline and taxane-based regimens, probably indicating adverse prognosis in terms of relapse-free and overall survival [4]. Oncologists are therefore pursuing more personalized therapies. Gene expression analysis has been widely incorporated in these studies, but less is known about epigenetic factors such as microRNAs (miRNAs) and their role in tailoring an individual systemic and surgical approach for breast cancer patients [5, 6]. MiRNAs are a regulatory class of small non-coding RNAs, approximately 20–23 nucleotides in length that have been described to post-transcriptionally modify gene expression. The deregulation of certain miRNAs has been associated with carcinogenesis in form of oncogenes, while others act as tumor suppressors [7].

## Aim

The aim of the study was to examine selected miRNAs in TNBC core biopsies sampled before preoperative chemotherapy and the subsequent pathologic response in mastectomy or breast conservation specimens.

## Patients and methods

The study was conducted under Institutional Review Board protocol # RNN/226/11/KE/13/12/2011, Medical University of Lodz. All patients gave written informed consent. Prior to treatment, ultrasound-guided 14-gauge core needle biopsies using an ultra automatic biopsy instrument (Pro-Mag TM, Angiotech) were collected from 11 female patients with inoperable locally advanced breast cancer or large operable tumors suitable for down-staging, and from three healthy controls (breast reduction procedures) at the Cancer Center between December 2011 and April 2012. Four to five specimens were obtained from each lesion, half of which were frozen immediately at −80 °C, for subsequent miRNAs profiling. The other samples were paraffin embedded and reviewed by specialist breast pathologists in the Department of Pathology. Estrogen receptor (ER) and progesterone receptor (PR) status were determined by immunohistochemistry (IHC) using the Allred score.

Human epidermal growth factor receptor 2 (HER2) status was evaluated by IHC or by fluorescence in situ hybridization. Samples were considered ER/PR negative if less than 1 % of the tumor cells were immunoreactive. Samples were considered HER2 negative with IHC 1+ staining or with a score of 2+ and no HER2 gene amplification when tested by FISH. TNBC were defined as ER, PR, HER2 negative. TNM clinical staging was assessed by mammography, ultrasound of the breast, axilla, and abdomen, bone scan and chest X-ray. In selected cases, MRI of the breast was performed. The following preoperative chemotherapy regimens were used: AT (doxorubicin 50 mg/m^2^, docetaxel 75 mg/m^2^) in five patients, AC (doxorubicin 60 mg/m^2^, cyclophosphamide 600 mg/m^2^) in four patients, EC (epirubicin 75 mg/m^2^, cyclophosphamide 600 mg/m^2^)—in one patient and AC+T (doxorubicin 60 mg/m^2^, cyclophosphamide 600 mg/m^2^, docetaxel 75 mg/m^2^)—in one patient. Upon completion of chemotherapy (six cycles every 21 days, except for AC+T—eight cycles), specialist breast surgeons performed mastectomy or breast conservation, with axillary dissection or sentinel node biopsy. Pathologic response in the mastectomy or breast conservation specimens was assessed by specialist breast pathologists. pCR was defined as postoperative microscopic absence of invasive or in situ carcinoma in breast tissue, and axillary lymph nodes after preoperative chemotherapy. A near complete response with only minimal residual disease was described as small clusters of tumor cells in the primary tumor site or lymph node or minimal cellularity in the surgical specimen, with 90 % loss of tumor cells. Pathologic non-response (pNR) was defined as no change or only minor change in individual malignant cells, but no reduction in overall cellularity. Partial pathologic response (pPR) was defined as reduction in overall cellularity, not exhibiting the changes listed for pCR, near-pCR or pNR. pCR and near-pCR were key points in statistical analysis.

## MiRNA profiling

RNA was isolated using miReasy Mini Kit 50 (Qiagen). In all 11 TNBC core biopsies we analyzed 19 miRNAs per sample: hsa-miR-512-5p, hsa-miR-190a, hsa-miR-200b-3p, hsa-miR-122-5p, hsa-miR-346, hsa-miR-148b-5p, hsa-miR-449a, hsa-miR-191-5p, hsa-miR-203a, hsa-miR-577, hsa-miR-93-5p, hsa-miR-126-5p, hsa-miR-423-5p, hsa-miR-129-5p, hsa-miR-193b-5p, hsa-miR-182-5p, hsa-miR-136-5p, has-miR-222-5p and hsa-miR-135b-5p (Exiqon, Copenhagen, Denmark). We selected these miRNAs from the literature, miRNA database and breast cancer conferences. They represent various epigenetic pathways involved in migration, invasion, epithelial-mesenchymal transition, cancer dormancy, switch to the fast growing phenotype, drug resistance, etc. [8–18]. We also used unpublished information from miRNA in vitro profiling studies performed on breast cancer cell lines. For normalization of the data, we have applied the average of the hsa-miR-103a-3p and hsa-miR-107 as this was found to be the most stable normalizer.

## MiRNA real-time qPCR

10 ng RNA was reverse transcribed in 10 μl reactions using the miRCURY LNA™ Universal RT microRNA PCR, Polyadenylation and cDNA synthesis kit (Exiqon). Each RT was performed in duplicates. cDNA was diluted 100 × and assayed in 10 μl PCR reactions according to the protocol for miRCURY LNA™ Universal RT microRNA PCR; each microRNA was assayed once by qPCR on the microRNA Ready-to-Use PCR, Custom Pick-&-Mix panel. Negative controls excluding template from the reverse transcription reaction were processes and profiled similarly. Amplification was performed in a LightCycler^®^ 480 Real-Time PCR System (Roche, Basel, Switzerland) in 384-well plates. The amplification curves were analyzed using the Roche LC software, both for determination of Cp (by the second derivative method) and for melting curve analysis.

## Data analysis

Amplification efficiency was calculated using algorithms similar to the LinReg software. All assays were inspected for distinct melting curves and the Tm was confirmed to be within known specifications for the assay. It was important for assays to be detected with 5 Cp’s less than the negative control, and with Cp < 37 to be included in the data analysis. Data that failed to reach these criteria were excluded from further analysis. NormFinder was used to identify the optimum normalizer which was the average of assays detected in all samples (average − assay Cp).

## Statistical analysis

The Wilcoxon signed-rank test was used to compare related samples. As the study was considered preliminary and miRNAs tested were selected due to biologic function we did not adjust for multiple testing at this stage. Ingenuity pathway analysis (IPA) bioinformatic database was used to evaluate potential functional significance of differentially expressed miRNAs.

## Results

Patients enrolled in the study were aged between 31- and 81-years-old, mean age was 54.1 years. Histopathological tumor types were invasive ductal breast cancer (11 patients). Tumor grades were G3 in all patients. Tumor stage was: IIB—in four patients, IIIA—in six patients, and IIIB—in one patient. Receptor status was triple negative in all patients. After preoperative chemotherapy, pCR was not achieved, but near-pCR was noted in 27 % (3 out of 11) patients (Table 1). Statistical analysis showed that 3 of 19 miRNAs differed in relation to pathologic response. Among these miRNAs, we identified—miR-200b-3p (*p* = 0.0662) up-regulated, miR-190a (*p* = 0.0662) up-regulated and miR-512-5p (*p* = 0.0641) down-regulated.

**Table 1:** Clinicopathological characteristics of breast cancer patients

Patient	Age (years)	Race	Histological type	Grade	Receptor subtype	Clinical staging before preoperative chemotherapy	Preoperative chemotherapy	Pathological staging in postoperative specimen	Type of surgery
Pt1	31	White	IDC	G3	TN	cT1N2	AT	ypT1aN0 near-pCR	BCS
Pt2	36	White	IDC	G3	TN	cT2N2	AC+T	ypT0N2 pCR in breast, no response in axilla	Mastectomy
Pt3	44	White	IDC	G3	TN	cT3N1	AT	ypT1aN0 near-pCR	BCS
Pt4	45	White	IDC	G3	TN	cT3N0	AC	ypT2N0	Mastectomy
Pt5	47	White	IDC	G3	TN	cT3N0	AC	ypT2N0	Mastectomy
Pt6	55	White	IDC	G3	TN	cT3N1	AT	ypT1aN0 near-pCR	BCS
Pt7	55	White	IDC	G3	TN	cT2N1	AT	ypT2N1	Mastectomy
Pt8	55	White	IDC	G3	TN	cT3N0	AC	ypT2N0	Mastectomy
Pt9	66	White	IDC	G3	TN	cT4N1	AT	ypT3N3	Mastectomy
Pt10	80	White	IDC	G3	TN	cT2N2	EC	ypT1cN1	Mastectomy
Pt11	81	White	IDC	G3	TN	cT3N1	AC	ypT3N0	Mastectomy

*IDC* invasive ductal carcinoma, *TN* triple negative

Preoperative chemotherapy regimens: *A* doxorubicin, *E* epirubicin, *C* cyclophosphamide, *T* docetaxel, *BCS* breast conserving surgery

**Fig. 1:**
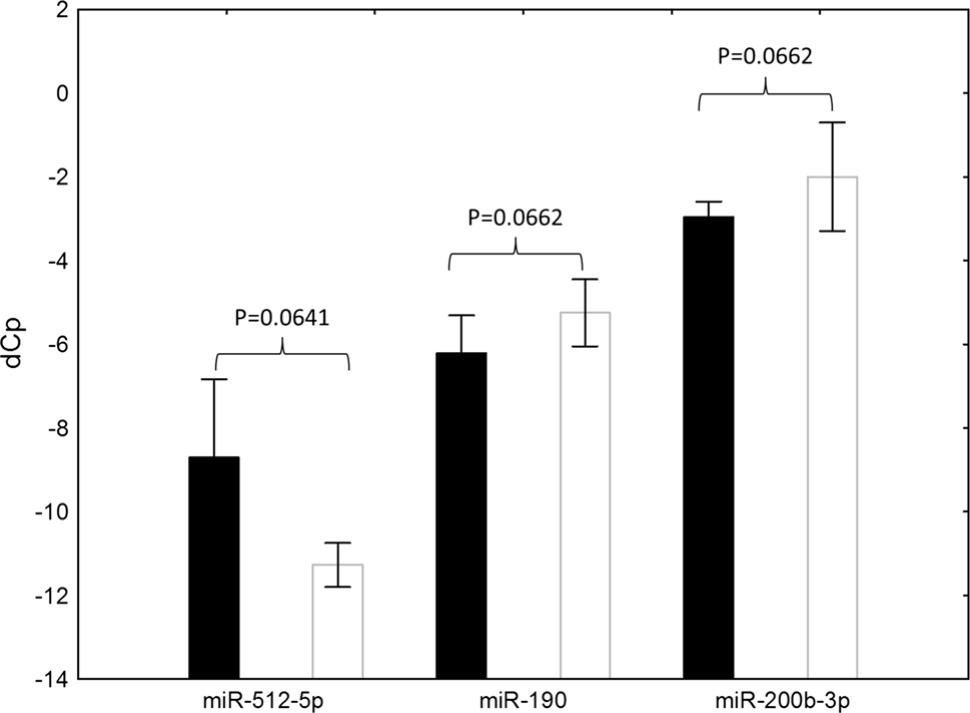
*Black bars* represent patients with poor prognosis, while *white bars* represent miRNA expression levels of individuals with near-pCR (good prognosis)

**Fig. 2:**
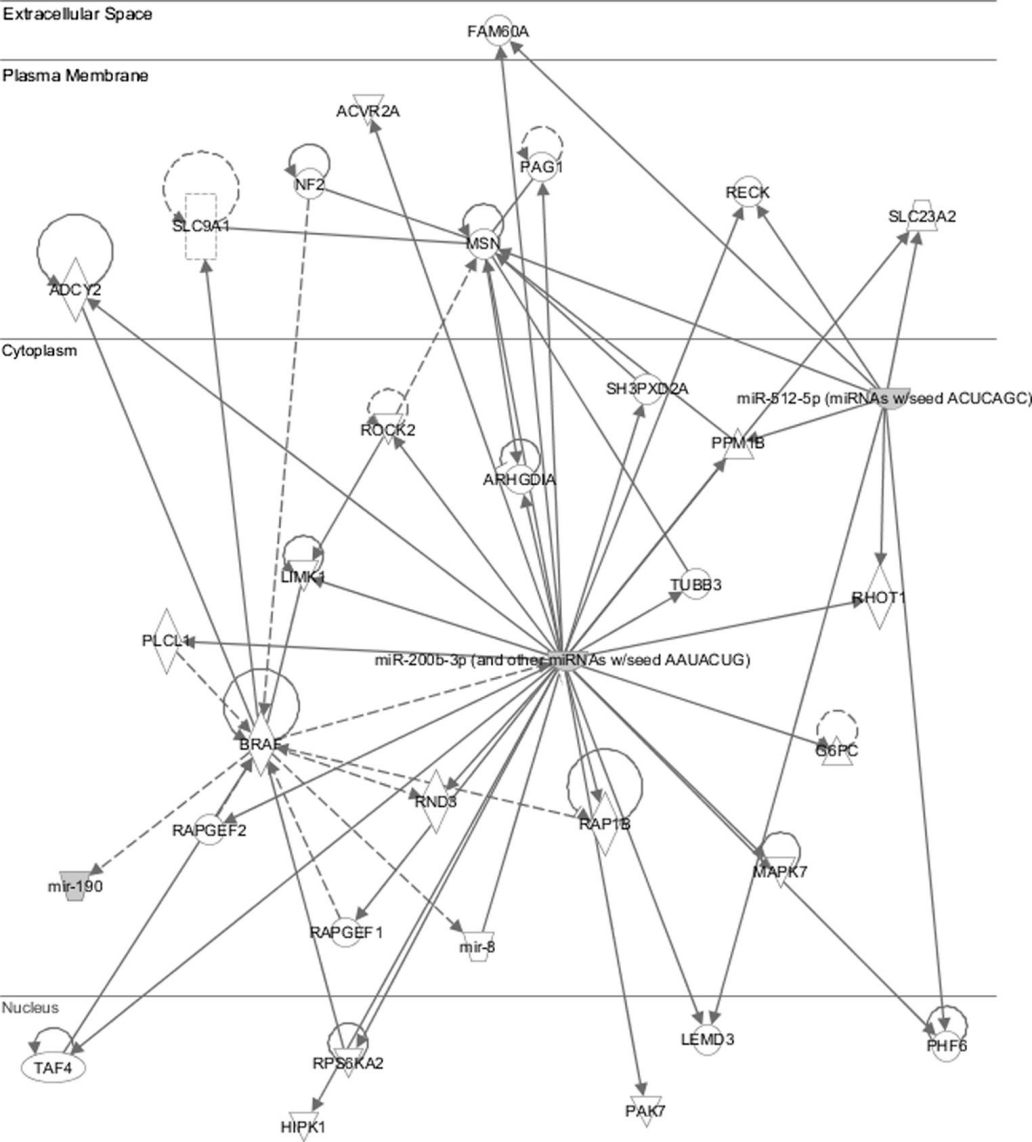
Functional significance of differentially expressed miRNAs—miRNA-512-5p, 190a and 200b-3p (Ingenuity pathway analysis). *Diamonds* represent enzymes, *triangles* represent kinases, *rectangles* represent ion channels, *trapezoid markers* represent membrane transporters, *inverted trapezoids* represent miRNAs, *circles* represent other types of molecules. *Arrows* represent direction and type of interaction according to Ingenuity pathway analysis standard manual (http://bioinfo.cnio.es/files/training/Functional_Analysis_Course/UBio_FuncAnalysis_Ingenuity.pdf)

*p* values of analyzed miRNAs and type of pathologic response, e.g. near-pCR (good response) versus non-near-pCR (poor response) are shown in Table 2 and Fig. 1. The three miRNAs with *p* values close to significance underwent pathway analysis using the IPA software. They were shown to be significantly linked to cellular assembly and organization/tissue development functional network with a −log10(*p*) of nine representing a strong non-random association (Fig. 2).

**Table 2:** Association of analyzed miRNAs and type of pathologic response (good vs. poor) in TNBC

miRNA	Good response average Cp	SD poor response	Poor response average Cp	SD good response	*p* value
hsa-miR-512-5p	−11.27201	1.870254	−8.702935	0.5255918	0.064078
hsa-miR-190a	−5.246738	0.9024946	−6.213683	0.8006026	0.066193
hsa-miR-200b-3p	−1.997353	0.370737	−2.960642	1.2972366	0.066193
hsa-miR-346	−9.852577	1.1360037	−9.914622	0.208277	0.182422
hsa-miR-148b-5p	−10.46267	0.5754232	−10.71966	0.2461868	0.220671
hsa-miR-449a	−11.08681	0.6922827	−11.00305	1.1720073	0.305059
hsa-miR-203a	−7.459372	1.0608366	−6.144586	2.7639684	0.379775
hsa-miR-577	−3.580436	0.3682042	−3.571102	0.4376185	0.438578
hsa-miR-93-5p	−0.017407	0.9109349	−0.546595	1.6692004	0.540291
hsa-miR-126-5p	−3.573845	1.4692401	−3.6294	0.3382967	0.540291
hsa-miR-423-5p	−3.367891	0.6627491	−3.099422	0.1176075	0.683091
hsa-miR-129-5p	−10.92072	0.9344205	−10.62819	0.84145	0.698535
hsa-miR-193b-5p	−8.425986	0.6266225	−7.939713	0.5464233	0.73244
hsa-miR-182-5p	−3.645599	1.0303241	−3.417605	1.793051	0.838257
hsa-miR-136-5p	−4.099501	1.5175184	−4.008906	0.1797492	0.838257
hsa-miR-135b-5p	−3.057166	1.638696	−3.151112	1.4110264	0.838257
hsa-miR-191-5p	−2.948123	0.337603	−3.021525	0.1843927	1
hsa-miR-122-5p	−12.42259	1.202344	−12.26785	0.3695887	1
hsa-miR-222-5p	−11.04475	0.9186428	−10.8786	1.0133855	1

## Discussion and conclusions

In the current study, we hypothesized that altered levels of selected miRNAs correlated with sensitivity to preoperative chemotherapy in TNBC patients. Numerous studies have sought to identify miRNA markers in the serum of cancer patients, but little is known about how miRNAs levels found in breast cancer samples relate to drug resistance [8]. Tryndyak et al. [9] have shown convincingly that transfection of breast cancer cell line MDA-MB-231 with miRNA-200b-3p inhibits epithelial-to-mesenchymal transition (EMT), reversing this aggressive phenotype to a milder form and increasing sensitivity to doxorubicin. Similarly, we used preoperative anthracyclines in all patients with large tumors, and 3 out of 11, with altered miRNA-200b-3p levels, responded well to treatment, with a tiny 2-mm residual cluster of viable cancer cells in the primary tumor bed, permitting breast conservation. Perhaps in view of the small sample size, there was no statistically significant difference, although we observed a trend to significance (*p* = 0.06) for the hypothesized correlation. Further research is needed and the present study should be considered to be preliminary. Pogribny et al. [10] have pointed out that miR-200b-3p was among the most deregulated miRNAs in the MCF-7 *cis*-platin resistant human breast adenocarcinoma cell lines. In our study none of the enrolled patients were treated with *cis*-platin, but based on the literature such regimens are efficacious in TNBC BRCA-mutation carrier breast cancer patients. However, in our study none of included patients were BRCA1/2 positive. Another miRNA whose expression differed between TNBC good and poor responders to anthracyclines and taxanes in our analysis was miR-190a (*p* = 0.0662, trend to significance).

Almog et al. have demonstrated in human breast carcinoma, glioblastoma, osteosarcoma and liposarcoma that over-expression of miRNA-190 governed the reverse switch of fast-growing angiogenic phenotype to the dormancy phase. These authors conducted their studies mainly in human cell lines, tissue cultures, mice and finally in 15 surgical specimens from patients with brain tumors [11, 12]. We have analyzed miR-190a expressions entirely in breast cancer patients, with reference to the database of in vitro experimental models published by other researchers.

Port et al. [13], who compared miRNAs expression patterns (e.g., miR-512-5p) in chemoresistant cancer cell lines, have emphasized that it would be of interest to examine tumor samples of patients with both chemosensitive and chemoresistant tumors to analyze whether the fluctuations, e.g. up- and down-regulations of selected miRNAs, are also found in vivo.

Statistical analysis in the current in vivo study have shown differences in miR-512-5p expression in relation to pathologic response in TNBC patients that failed to reach statistical significance, although a trend was observed (*p* = 0.064). Further studies in larger cohorts are needed on account of the complexity of epigenetic modulations, their interactions with genetic pathways and the difficulty of direct translation from laboratory to clinic. We used two bioinformatic programs TargetScan and PicTar to identify target genes of miRNA 190a, 200b-3p and 512-5p; e.g. 104 target genes for miRNA 190a and 97 target genes for miRNA 200b-3p were found by PicTar, 94 target genes for miRNA 512-5p by TargetScan. Examples of target genes: *BCL11A* (B cell CLL/lymphoma 11A), *CALCR* (calcitonin receptor), FOXP2 (forkhead box P2), *HOXC5* (homeobox C5) for miRNA 190a; *PLCB1* (phospholipase C, beta 1), *MYCN* (v-myc myelocytomatosis viral related oncogene), *CCND2* (cyclin D2), RERG (RAS-like, estrogen-related growth inhibitor) for miRNA 200b-3p; *BCL2L2* (BCL2-like 2), *POLD3* polymerase (DNA-directed delta 3, accessory subunit), *c*-*Myc* for miRNA 512-5p, etc. MiRNA and gene network is shown in Fig. 2 [12].

A key issue of neoadjuvant trials is pCR, as a surrogate end point, which strongly correlate with long-term survival in TNBC [4]. In our analysis none of 11 TNBC patients achieved pCR, but in three patients a good response to chemotherapy (near-pCR) was noted. Symmans et al. [14] have concluded in their study that minimal residual disease (RCB-I, residual cancer burden I according to M.D. Anderson criteria) in 17 % of patients carried the same prognosis as pCR (RCB-0). Moreover, von Minckwitz et al. have demonstrated that Ki67 measured in residual hormone-receptor negative tumors can further subdivide this unfavorable group of patients without a pCR. High Ki67 levels in residual disease predict a considerable risk of relapse, but patients with low Ki67 levels showed a comparable outcome to patients with a pCR for disease-free and overall survival [15]. In all three TNBC patients who achieved near-pCR in our study very low Ki67 levels (6 %) were measured.

In summary, our results indicate that higher miR-200b-3p, higher miR-190a and lower miR-512-5p expression levels in core biopsies sampled from TNBC patients may be associated with better pathologic response to chemotherapy and the increased feasibility of breast conserving surgery in these patients. Although these results were from a small cohort, they provide an important basis for larger, prospective, multicenter studies to investigate the potential role of miRNAs not only in breast cancer cells, but also in adjacent tissues and serum as predictive biomarkers. More precise identification, before initiation of treatment, of those patients who would benefit from specific chemotherapeutic regimens may improve response rates, avoid toxicity of ineffective therapy and guide the extent of necessary surgery i.e. breast conservation versus mastectomy [16–18].
